# Households Contrasted

**Published:** 1882-04

**Authors:** 


### HOUSEHOLDS CONTRASTED.

A GORGED anaconda is the mildest animal in nature. You may kick him until you are tired, and he won't take the trouble to raise his head or give a hiss. We have remembrances as fresh as of yesterday, of going out to the pig-pen of a frosty fall morning, and witnessing the changing mood of the occupants while waiting for the men to throw in the corn. How they would squeal, and grunt, and bite, and snap at each other! these modes of expression becoming "excelsior" in geometrical progression with each passing minute of delay. With the first mouthful, "order reigns in Warsaw." A few minutes later, a wag of delight takes hold of the tail, and at the "closing stretch," pig spoons to pig, and side by side they while their time away in delicious, dozy grunts. Pigs and people are not far apart. There are men, and—shall we say it?—women, too, who, before they get their breakfast in the morning, are as ferocious as she-tigers. They have brutalized themselves by late dinners, or over-hearty suppers the preceding evening, or they have sat up until midnight, or later, in doing what they considered indispensable—working, it may be in actual weariness and suffering, with the effect, that when the morning comes, they are not rested, not refreshed, and, as a consequence, the nervous system is deranged; and if they are up soon enough for family worship, they come into the room where glad children and servants (for both children and servants being healthy, are light-hearted in the mornings) are all in waiting, with a tremendous scowl on the countenance, or a sheepish, slovenly indifference, according to the temperament of the human animal. But not the honey droppings of the "Word;" not the glad songs of praise from other lips; or thanks for the nightly deliverances or the morning mercies, the bright sunshine, the dancing fire on the hearth, while the frost is at the window; not the consideration that every child is the picture of health, and a well-spread board, white and clean and smoking and abundant, is ready, without even the trouble of ordering; not all these considerations are sufficient to mellow down that ugly nature, but it drawls along to the table to whine and complain and growl and fret. If a child happens to laugh outright in its gladness, there is an instantaneous snap at it, if, indeed, it is not driven from the table, or slapped over with the hand. If the toast is not browned to a turn, or the steak is the tiniest bit over or under-done, the cook is raved at with the savageness of a polar bear. If the table-maid happens in her haste to trip a toe or let fall a roll, noise enough is made about it to fill a barn; and if a child overturns a cup, a hyena comes up in the "dissolving view."

Reader! what think you? does this picture of a daily reality in more households than a dozen, suit you in whole, or even small part? Be counseled in time, that one of two results will fall to your lot: if, in humility and repentance towards God and man, you do not promptly seek a remedy against so low a crime, you will either die prematurely, or end your days in an asylum, if not in a worse place still. If you begin a day in so ungodly a manner, it is a "bad beginning" for you; the whole of that day will be a cloud; the night will set in with humiliating or fierce remorses, and it will be a day lost—lost forever. If it were only lost to you, it is of comparatively small moment, except to yourself. But that is not all. You clouded over a sunshine which God had sent to gladden the members of your family. You robbed them of God's good gift.

**HEALTH OF CITIES.**—Estimating New-York to contain a million of people, and London two millions and a half, five hundred and fifty dying every week on an average in New-York and twelve hundred and twenty-five in London, the mortality of the two great commercial centers of the Old and the New World is just the same. But there is no city in the civilized world so admirably adapted for health as New-York, bounded as it is on two of its three sides by large rivers, while its topical formation is such that it has an almost perfect drain from its longitudinal center to the rivers. A proper attention to two things would make it exceed any great city in healthfulness. First, a more perfect system of street-cleaning. Second, a better class of dwellings for the poor, combining perfect ventilation and cleanliness.

**BEAUTIFUL HAIR**—Finds worshipers the world over. When it is abundant and tastefully adjusted, it "sets off" the face of beauty, and may be made to soften even a deformity. Its arrangement exhibits the taste of the wearer, and it may be made more ornamental than the richest jewel ever dug from Golconda's mines. But it is a jewel not found in the casket of one woman in a thousand. The hair should be cultivated from infancy, by keeping the head clean and cool night and day; it should be worn as short as a boy's until the thirteenth year is completed; a comb, or a pin, or a tie should not be allowed for an hour, nor a "parting" nor a braid; nothing should ever touch it but a tortoise or fine rubber-comb and pure soft water; it should never be allowed to bear more than its own weight, nor detained from its natural direction; if any thing, it should be supported unstrained. Ventilation and cleanliness, from infancy to budding womanhood, are essential to having hair that is thick, long, luxuriant, permanent, and glistening with life. Fashion, all blind and remorseless as she is but too often, is for once philosophical and wise in introducing nets to hold the hair of girls at the neck behind.

The pernicious metallic hair-pins have "killed" the hair of our wives and daughters, whose entire stock in trade, that is their own, scarcely equals in size a common hickory-nut. It has been cut by the harsh hair-pin, pulled from its roots by braiding and tying, and actually rotted at its origin by an imbedded mass of grease, dust, and dandruff, stopping up every pore, preventing exhalation, confining the heat, and setting up a permanent and destructive inflammation about every bulb. A clean scalp and pure soft water are the best pomatums in the world for man or woman, boy or girl, young or old.

COLD FEET.—It is impossible to have vigorous health if the feet are habitually cold; no amount of external covering can keep them warm. Wearing pepper and other irritants in the stockings, is generally inefficient, is always hurtful in its tendencies, and never accomplishes a permanent radical good. One of the most uniformly efficient means of keeping the feet warm is to wash them in water at least as cold as the atmosphere of the room, night and morning; let it be done within a minute in very cold weather, then wipe and rub them rapidly and thoroughly with a very coarse towel, dress, and when practicable, take a walk, or dry them by the fire, rubbing them well with the hands.

In addition, let half an inch of curled hair be basted to a piece of cloth and slipped in the stocking, the hair touching the soles of the feet to titillate the skin, and thus aid in drawing the blood thither to warm them. The hair conducts the moisture from the feet to the woolen cloth, and thus keeps them dry. These hair-soles should be placed before the fire at night, so as to be thoroughly dried by the morning. Cork-soles absorb moisture from the shoe and the feet also, and require several days to be thoroughly dried. India-rubbers confine the dampness about the feet, hence they should be promptly removed as soon as the wearer ceases walking, nor should they be used except in muddy, slushy weather.

### THROAT AIL CAUSES.

A RESPECTED correspondent writes: "I took cold three weeks ago, preaching in a draft. It affected my throat and ears also, so that in speaking I was annoyed by a jingling kind of sound. I was foolish in taking it as I did." We replied in part, "Preaching in the draft has destroyed many a valuable clergyman. You should make up your mind never to do it again. Stand in the corner of the room. Is it likely that the effect of any given sermon is worth a minister's life, when he otherwise might have lived twenty years longer, with the chances of repeating this effect thousands of times? Why don't you preachers study ecclesiastical economy more?"

We must say that clergymen many times act as if they were made out of steel, as if their health was impregnable, and that nothing could effect it injuriously. They ought to remember that they have no charmed lives, and that their only secure shield against the shafts of disease and death, which fly around all at every step, is a RATIONAL CARE.

---

### TO BE SAFE,

BE in proper places at proper times, and mind your own business. With such restrictions, we believe human life is as safe in New York as in any other city on the globe. Nine times out of ten, the reports of persons who have fared badly, or who have fallen into the hands of the Philistines, indicate one of two things—an out-of-the-way place, or an unseasonable hour. A young man attends a private party, leaves at two o'clock in the morning, and is heard of no more. Another visits New York, and, with his pockets full of money, promenades the river streets alone, an hour or two after dark. It is at one o'clock in the morning that a man was "*knocked down, and robbed of several thousand dollars,*" with no trace of the robbers—unless you get him to tell where and what he was doing between the hours of decent bed-time and those of early morning. The fact is, a good many of the robberies fathered on large cities never took place. The assignation and the gaming table are the maelstroms of half the "lost pocket-books" of the city morning newspapers.

### BE CAREFUL.

Be careful, young man, when you first commence business, to choose such an occupation, as will be the most conducive to health; for it will be more valuable to you than silver or gold. Be careful, young lady, how you trifle with life or death, as though they were of no value; the course you are pursuing will endanger them in a great many ways. You often, in damp weather, promenade the street, with thin soled shoes, regardless of the consequences; besides, you attend balls and parties, in winter, wearing that kind of apparel which is only appropriate for summer. Then, if you exercise, so as to be a little too warm, you forget to *be careful*, and often sit or stand in a draught of cold air, until perspiration is checked—then the "bad cold," often leading to a "fatal disease." Add to this, late hours from novel reading, and late rising, too, which prevents the morning breeze from blowing gently around you, to give you new life and animate your depressed spirits. It's true, you may go on in this way for months, and perhaps for a few years: but remember, the day of reckoning will come; then you would retract, but it may be too late; for by this time the "hollow cough" begins to startle you; but you indulge the hope that it's only a "cold," and form the resolution to be more careful for the future. But what is past can never be recalled. Your disease may now baffle the skill of the most eminent physicians. Despite the ceaseless watchings and prayers of a kind-hearted father and an affectionate mother, the king of diseases performs his task; your form becomes almost a shadow, and you sink into a premature grave. But how often disease might be prevented! if we would *be more careful*, and attend to the laws of health. While penning these lines, we are carried by retrospection to the days of our youth, when, if we had been guided more by judgment, we might now be enjoying better health. But like many others, we are now trying to *be careful*, and to profit by the advice we receive from our physician. Let us hope that the subject of health, at no distant day, will be looked at in its true light, and receive the attention it deserves and demands, and not be so often sold for a few transitory pleasures.

